# Improving Vaccine Clinic Efficiency Through the CANImmunize Platform

**DOI:** 10.2196/53226

**Published:** 2024-10-16

**Authors:** Ananda Kuatsidzo, Kumanan Wilson, Sydney Ruller, Blake Daly, Roland Halil, Daniel Kobewka

**Affiliations:** 1 Department of Medicine, The Ottawa Hospital, Ottawa, ON, Canada; 2 Clinical Epidemiology Program, Ottawa Hospital Research Institute, 1053 Carling Ave, Ottawa, ON, K1Y4E9, Canada, 1 613-737-8899 ext 15935; 3 Bruyère Research Institute, Ottawa, ON, Canada; 4 Department of Family Medicine, Bruyère Academic Family Health Team, Ottawa, ON, Canada

**Keywords:** digital solutions, vaccine, CANImmunize platform, CANImmunize, platform, Canada, Canadian, workflow, booking, health care, digital health, hospital, patient, personnel

## Abstract

Our objective was to evaluate the CANImmunize digital solution and measure the impact on workflow and appointment booking at Bruyère Hospital.

## Introduction

Running immunization clinics in any organization or health system requires detailed planning, coordination, and collaboration. Barriers such as long wait times, misinformation about vaccination benefits, operating hours and locations of clinics, and inconsistent methods to record vaccination status contribute to lower vaccination rates [1,2].

CANImmunize Inc is a Canadian technology company that created a digital solution for end-to-end digital vaccine management that permitted centralized web-based booking across multiple clinics; digital preconsent; clinic management; and provision of proof of vaccination records, including integration with provincial registries and supported adverse event reporting systems.

Vaccine clinics are typically paper based, which requires significant human resources. Paper-based data collection is time-consuming, limiting the number of vaccinations that can be administered and making it difficult to track vaccination rates [3]. Our objective was to evaluate the CANImmunize product and measure the impact on workflow and appointment booking.

## Methods

### Setting and Participants

We implemented CANImmunize between November 2020 and April 2021 at Bruyère Hospital in Ottawa, Ontario. We offered vaccination appointments to staff, their families, and Bruyère Academic Family Health Team primary care clinic patients. All staff and patients were sent information about how to download the app, create an account, and sign up for an appointment.

### Digital Solution

CANImmunize software consists of three components—the CANImmunize web portal, ClinicFlow, and the CANImmunize app. The web portal allows patients to book their appointment, complete COVID-19 screening, provide consent for the flu vaccine, and subsequently upload the vaccine receipt into Bruyère’s Occupational Health and Safety System. ClinicFlow allows immunizers to handle appointment scheduling by tracking appointments, cycle times, wait times, and the total number of appointments per day. The CANImmunize app provides a permanent record of all patients’ immunizations.

### Outcomes

During ClinicFlow implementation, we measured the number of vaccinations, appointments, and staff subscriptions to the CANImmunize app and the time spent per appointment. To determine changes in the number of staff vaccine appointments and time spent by immunization clinic staff per appointment, we used data from the year before ClinicFlow was implemented. We used staff hourly rates to calculate the cost savings per vaccination.

### Ethical Considerations

Our study qualifies as quality improvement because we report aggregate-level data from an implementation where the primary purpose was to monitor, evaluate, or improve the quality of services delivered. Therefore, our study was not reviewed by a research ethics board.

## Results

Over the study period, 1286 appointments were booked, and 2213 vaccines were administered to staff and their families. Appointments could have ≥1 person; for example, 1 staff member and their 2 children would be 1 appointment that results in 3 vaccines being administered. Each vaccine administrator reported a reduction in the time required for vaccination administration, decreasing from 15 minutes with the paper-based format to 10 minutes with the digital platform, resulting in total time savings of 107.2 hours (1286 appointments × 5 min).

Vaccine clinic staff reported a reduction in the clerical time required to upload staff vaccine data to the database, decreasing from 5 minutes per staff to 0 minutes, resulting in clerical time savings of 79.3 hours (952 staff × 5 min = 4760 min). In total, 952 Bruyère staff were vaccinated, with 174 individuals signing up for CANImmunize after their immunization appointment.

## Discussion

### Principal Findings

Productivity improved with time and cost savings after CANImmunize implementation. Booking through the app, completing consent forms before visits, easy patient registration, and automatic vaccination record uploading saved staff time and money. Automated record uploading improved the accuracy of vaccination rate tracking.

Consistent with our results, the World Health Organization described digital health as a safe and cost-effective way to use IT to improve health care access [4]. During the COVID-19 pandemic, several booking systems launched globally, becoming increasingly important as the complexities of the vaccine schedule increased due to the need for multiple doses and boosters. Moving forward, digital solutions to health problems will become increasingly important [5,6]. Vaccines will continually be promoted and administered to older populations where uptake has been historically low [7-9]. Therefore, well-developed software that can facilitate vaccine administration is essential.

Key lessons learned during our pilot included the importance of scheduling and family bubbles for improving throughput efficiency while maintaining social distancing, as well as the importance of ease of use among non–technically proficient individuals. These elements were incorporated into the broader population-wide release of the platform and contributed to its relative success.

### Conclusions

Digital vaccine clinic booking in a health care facility improved efficiency while facilitating accurate and comprehensive recordkeeping.
